# Prognostic value of radiation treatment time deviation in nasopharyngeal carcinoma receiving intensity-modulated radiotherapy

**DOI:** 10.1016/j.isci.2026.117124

**Published:** 2026-08-13

**Authors:** Wenxi Wu, Chao Li, Zongwei Huang, Ying Li, Xinyi Hong, Xiaoyong Liu, Yuxing Yu, Peidong Ou, Sufang Qiu, Fengjie Lin

**Affiliations:** 1 Clinical Oncology School of Fujian Medical University, Fujian Cancer Hospital, NHC Key Laboratory of Cancer and Metabolism, Fuzhou, China

**Keywords:** nasopharyngeal carcinoma, radiation therapy, chemotherapy, continuous interruption, survival

## Abstract

This study examines the prognostic impact of radiotherapy interruptions (RTIs) on nasopharyngeal carcinoma (NPC), comparing outcomes between continuous and interrupted radiotherapy (RT). A retrospective cohort of 2,059 patients with NPC treated with intensity-modulated radiotherapy (IMRT) from 2017 to 2019 was analyzed. Survival outcomes were assessed using Kaplan-Meier curves and log rank tests. Recursive partitioning analysis (RPA) determined optimal thresholds for radiation treatment time (RTT) and RTT deviation (ΔRTT). Univariate and multivariate Cox proportional hazards models identified independent prognostic factors for overall survival (OS), progression-free survival (PFS), locoregional failure-free survival (LRFFS), and distant metastasis failure-free survival (DMFFS). RPA established RTT and ΔRTT thresholds of 50 and 7 days, respectively; exceeding either threshold was associated with significantly inferior outcomes across all endpoints. Multivariate analysis confirmed ΔRTT as an independent prognostic factor for all endpoints (*p* < 0.05), with a significant interaction observed between ΔRTT and interruption frequency. In the IMRT era, ΔRTT ≥7 days is a critical adverse prognostic determinant in NPC; multiple RT interruptions further compromise survival, particularly with prolonged treatment duration.

## Highlights

• Critical survival impact of RTI in NPC treated with IMRT
• ΔRTT identifies a broader range of high-risk patients compared to traditional static metrics
• ΔRTT≥ 7 days and recurrent interruptions predict poor NPC survival outcomes

## Graphical abstract

**Figure:**
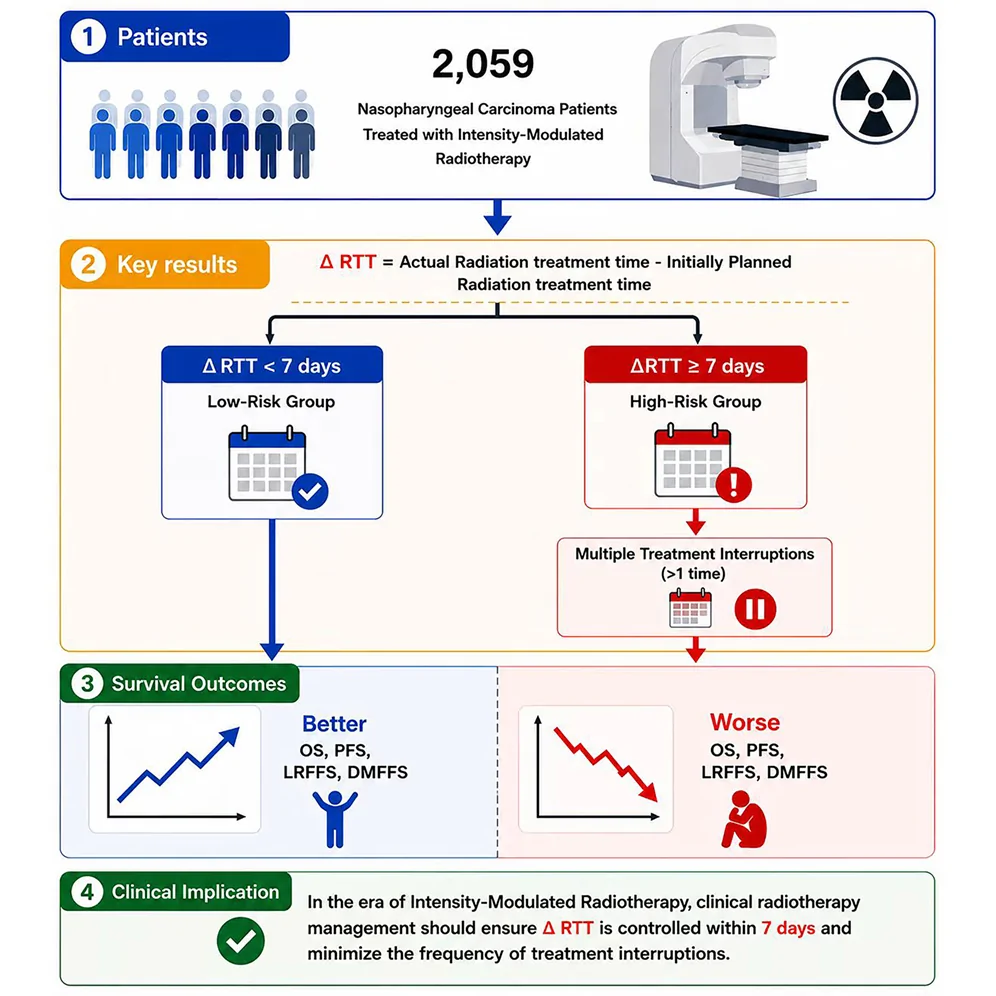

## Teaser

Oncology; Therapeutics

## Introduction

Nasopharyngeal carcinoma (NPC) demonstrates a pronounced ethnic and geographical distribution, characterized by significantly elevated incidence rates in Southern China, Southeast Asia, and the Middle East/North Africa regions.^1^^,^^2^^,^^3^ Within these regions, Fujian Province in Southern China is particularly affected. The etiology of NPC is complex, involving a multifactorial interplay of genetic predisposition, viral infections—primarily Epstein-Barr virus (EBV)—and various environmental factors, all of which are crucial to its pathogenesis.^4^^,^^5^^,^^6^ Despite significant advances in diagnostic and therapeutic methods, NPC remains a formidable challenge due to its unique anatomical location, early tendency for lymphatic metastasis, and proclivity for distant dissemination.

Radical radiotherapy (RT) is the standard treatment for early-stage NPC and a key component of the multidisciplinary management of locally advanced cases.^7^^,^^8^^,^^9^ Compared to traditional two-dimensional or three-dimensional radiation therapy, intensity-modulated radiotherapy (IMRT) delivers higher radiation doses more precisely to NPC while protecting adjacent critical structures and reducing treatment-related toxicity. IMRT enhances local control and survival rates in patients with NPC.^10^^,^^11^ However, its therapeutic effectiveness can be compromised by interruptions in the treatment schedule. These interruptions may result from equipment malfunctions, patient-related issues (e.g., RT intolerance or non-compliance), institutional factors (e.g., scheduling conflicts), or public holidays.^12^^,^^13^ The accelerated repopulation of tumor cells, particularly in rapidly proliferating tumors such as head and neck cancers, provides a radiobiological basis for the poor prognosis associated with extended treatment durations.^14^^,^^15^

Research has investigated radiotherapy interruptions (RTIs) in various tumors, including lung, breast, and anal squamous cell carcinoma, revealing a correlation between RTIs and unfavorable survival outcomes.^16^^,^^17^^,^^18^ For instance, prolonged RT has been associated with reduced overall survival (OS) in patients with stage III non-small cell lung cancer undergoing concurrent chemoradiotherapy (CCRT).^9^ Similarly, Yao et al. found that RTIs exceeding five days negatively impacted the prognosis of patients with stage T3-T4 NPC.^19^ Despite these findings, the precise influence of RTIs on survival outcomes in patients with NPC receiving IMRT therapy is still insufficiently examined. Additionally, research on the critical thresholds at which RTIs become detrimental to survival is inconsistent. In the context where IMRT has been widely applied in the treatment of NPC, more data are necessary to establish definitive RTI thresholds that could inform treatment strategies and improve patient outcomes.

Therefore, within the current backdrop, an in-depth evaluation of the impact of RTI on survival outcomes in patients with NPC receiving IMRT holds significant clinical importance for optimizing clinical RT protocols and enhancing patients’ quality of life. This study aims to conduct a comprehensive retrospective analysis of patients with NPC receiving IMRT, focusing on assessing the impact of RTI on survival outcomes, and to further compare the differences between continuous and interrupted RT on patient survival prognosis, in order to provide evidence-based support for developing more precise treatment implementation strategies in clinical practice.

## Results

### Clinical characteristics

Between January 2017 and December 2019, 2,059 patients met the study’s inclusion criteria. The median age of the cohort was 49 years (IQR, 41–56 years), including 1504 males and 555 females. The median follow-up duration was 55.57 months (range: 44.83–65.57 months), with 86.79% of patients being monitored for a period exceeding three years. The median of RTT was 46 days (IQR, 45–49 days). Clinical characteristics were stratified by median RTT into two groups: those with RTT longer than 46 days and those with RTT of 46 days or less (see Table 1). Patients exhibiting advanced TNM stages (either advanced T or overall stage) were more prone to experience extended RTT (*p* < 0.001 for each comparison). Moreover, patients who received 32–36 fractions tended to have a longer RTT (*p* < 0.001). In addition, significant differences were also observed in treatment approaches (*p* < 0.001). Another factor demonstrating significant variation was HGB levels (*p* < 0.05 for all; refer to Table 1).

**Table 1:** Baseline patient characteristics according to RTT

Characteristic	No. (%) of patients by RTT in days		Total	P
	≤46(*n* = 896)	>46(*n* = 1163)	(*N* = 2059)	
Gender	–	–	–	0.147
Male	640(71.43)	864(74.29)	1504(73.05)	–
Female	256(28.57)	299(25.71)	555(26.95)	–
Age, year	–	–	–	0.766
≤45	337(37.61)	430(36.97)	767(37.25)	–
>45	559(62.39)	733(63.03)	1292(62.75)	–
T stage (8th edition)	–	–	–	< **0.001**
T1	209(23.33)	169(14.53)	378(18.36)	–
T2	188(20.98)	137(11.78)	325(15.78)	–
T3	367(40.96)	506(43.51)	873(42.40)	–
T4	132(14.73)	351(30.18)	483(23.46)	–
N stage (8th edition)	–	–	–	0.394
N0	74(8.26)	87(7.48)	161(7.82)	–
N1	347(38.73)	432(37.15)	779(37.83)	–
N2	293(32.70)	422(36.29)	715(34.73)	–
N3	182(20.31)	222(19.09)	404(19.62)	–
Overall stage (8th edition)	–	–	–	< **0.001**
I	28(3.13)	23(1.98)	51(2.48)	–
II	144(16.07)	78(6.71)	222(10.78)	–
III	420(46.88)	518(44.54)	938(45.56)	–
IV	304(33.93)	544(46.78)	848(41.19)	–
EBV DNA titer, copies	–	–	–	0.094
negative	624(69.64)	849(73.00)	1473(71.54)	–
positive	272(30.36)	314(27.00)	586(28.46)	–
HGB, g/L	–	–	–	< **0.05**
<113	224(25.00)	304(26.14)	528(25.64)	–
113–151	619(69.08)	822(70.68)	1441(69.99)	–
>151	53(5.92)	37(3.18)	90(4.37)	–
Prescribed fractions	–	–	–	< **0.001**
28–31	254(28.35)	51( 4.39)	305( 14.81)	–
32–36	642(71.65)	1112(95.61)	1754(85.19)	–
Treatment modality	–	–	–	< **0.001**
RT alone	53(5.92)	52(4.47)	105(5.10)	–
IC + RT	185(20.65)	308(26.48)	493(23.94)	–
IC + RT + AC	29(3.24)	23(1.98)	52(2.53)	–
CCRT	81(9.04)	60(5.16)	141(6.85)	–
CCRT+AC	14(1.56)	11(0.95)	25(1.21)	–
IC + CCRT	469(52.34)	637(54.77)	1106(53.72)	–
IC+CCRT+AC	65(7.25)	72(6.19)	137(6.65)	–
Residual or not	–	–	–	0.651
No	732(81.70)	941(80.91)	1673(81.25)	–
Yes	164(18.30)	222(19.09)	386(18.75)	–
NLR	–	–	–	0.913
Normal	457(51.00)	596(51.25)	1053(51.14)	–
Abnormal	439(49.00)	567(48.75)	1006(48.86)	–
Number of IC sessions	–	–	–	< **0.001**
0	148(16.52)	123(10.58)	271(13.16)	–
≤3	448(50.00)	603(51.85)	1052(51.04)	–
>3	300(33.48)	437(37.58)	737(35.79)	–

RTT, radiotherapy treatment time; EBV-DNA, plasma Epstein-Barr virus (EBV) DNA; HGB, hemoglobin; RT, radiotherapy; IC + RT, radiotherapy following induction chemotherapy; CCRT, concurrent chemoradiotherapy; IC + CCRT, induction chemotherapy plus concurrent chemoradiotherapy; NLR, neutrophil-to-lymphocyte ratio; AC, adjuvant chemotherapy; IC, induction chemotherapy. ∗ Percentages may not add up to 100 due to rounding.

### Improving RTT and survival

**Figure 1:**
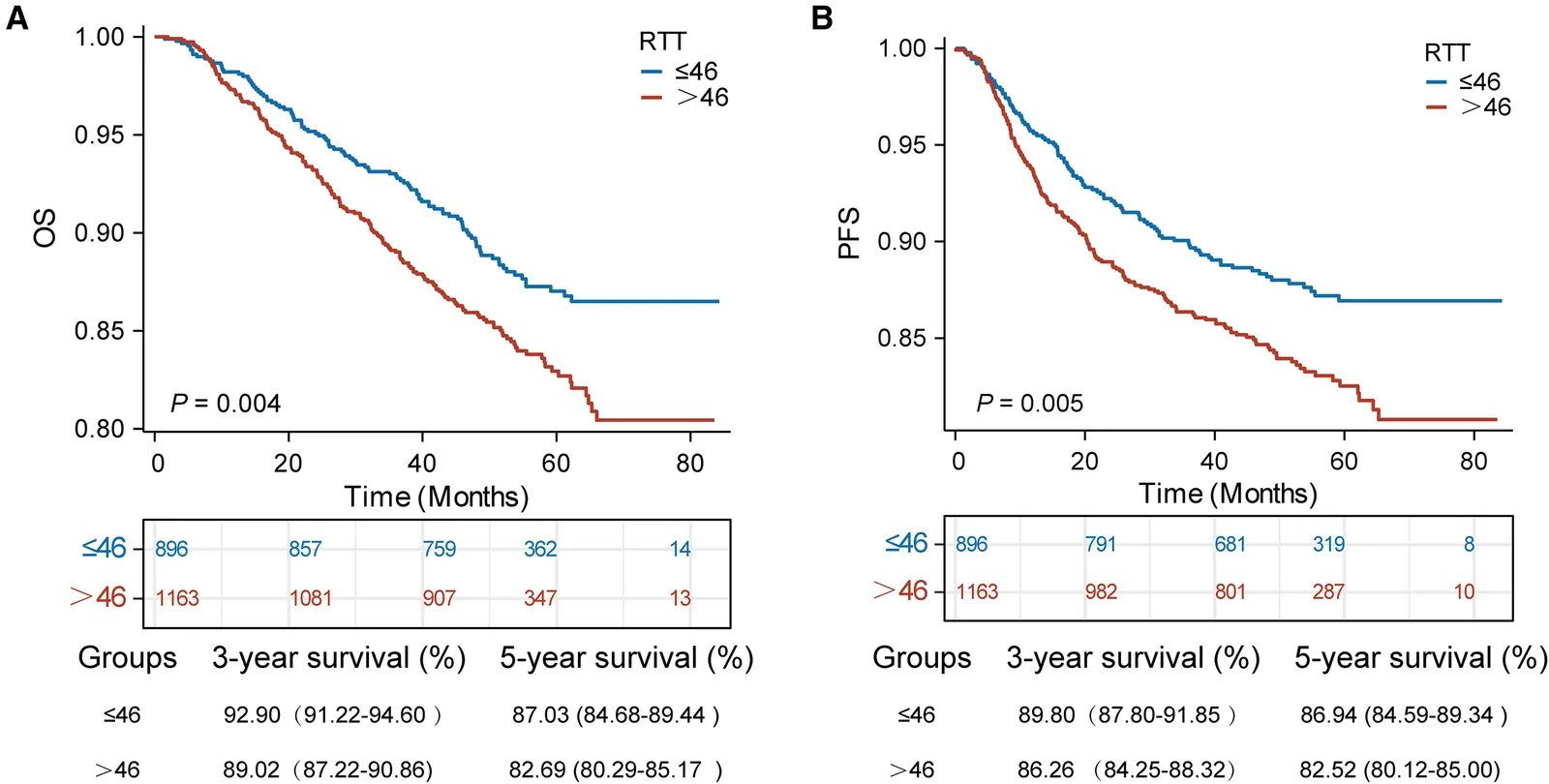
Kaplan-Meier’s plots showed (A) OS, (B) PFS divided by the RTT of ≤46 days, and >46 days, respectively. Abbreviations: RTT, radiotherapy treatment time; OS, overall survival; PFS, progression-free survival.

Patients with an RTT of 46 days or less had a five-year OS rate of 87.03%, while those with an RTT exceeding 46 days had a rate of 82.69%. Similarly, the five-year progression-free survival (PFS) rate was 86.94% for RTT ≤ 46 days, versus 82.52% for RTT > 46 days. The Kaplan-Meier curve analysis indicated that patients with RTT > 46 days had worse outcomes in OS, PFS, LRFFS, and DMFFS compared to those with RTT ≤ 46 days (*p* < 0.01, Figure 1; Figure S1). Univariate analyses revealed that an RTT exceeding 46 days was a significant predictor of an increased mortality risk and disease progression risk compared to an RTT of 46 days or less (*p* < 0.01, Tables S1 and S2).

**Figure 2:**
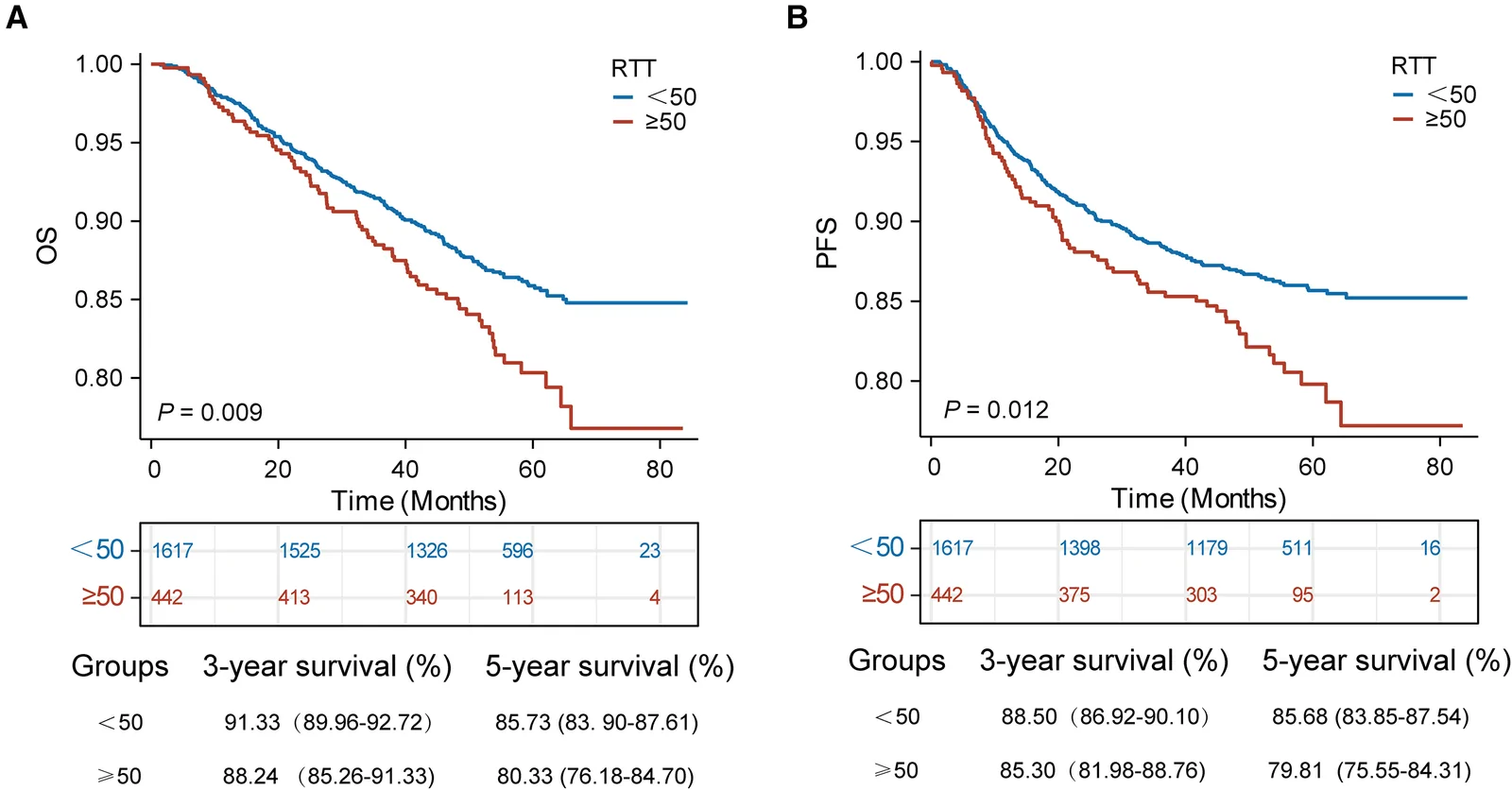
Kaplan-Meier survival curves illustrated (A) OS, (B) PFS categorized by the RTT of < 50 days and ≥50 days. Abbreviations: RTT, radiotherapy treatment time; OS, overall survival; PFS, progression-free survival.

To determine the optimal RTT threshold for predicting significant survival differences, RPA was conducted. The optimal threshold identified was 50 days. When RTT was adjusted to this threshold, patients with RTT ≥ 50 days exhibited significantly worse outcomes in OS, PFS, LRFFS, and DMFFS compared to those with RTT < 50 days, as reflected in the Kaplan-Meier curves (Figure 2; Figure S2).

### Survival of patients stratified by ΔRTT

**Figure 3:**
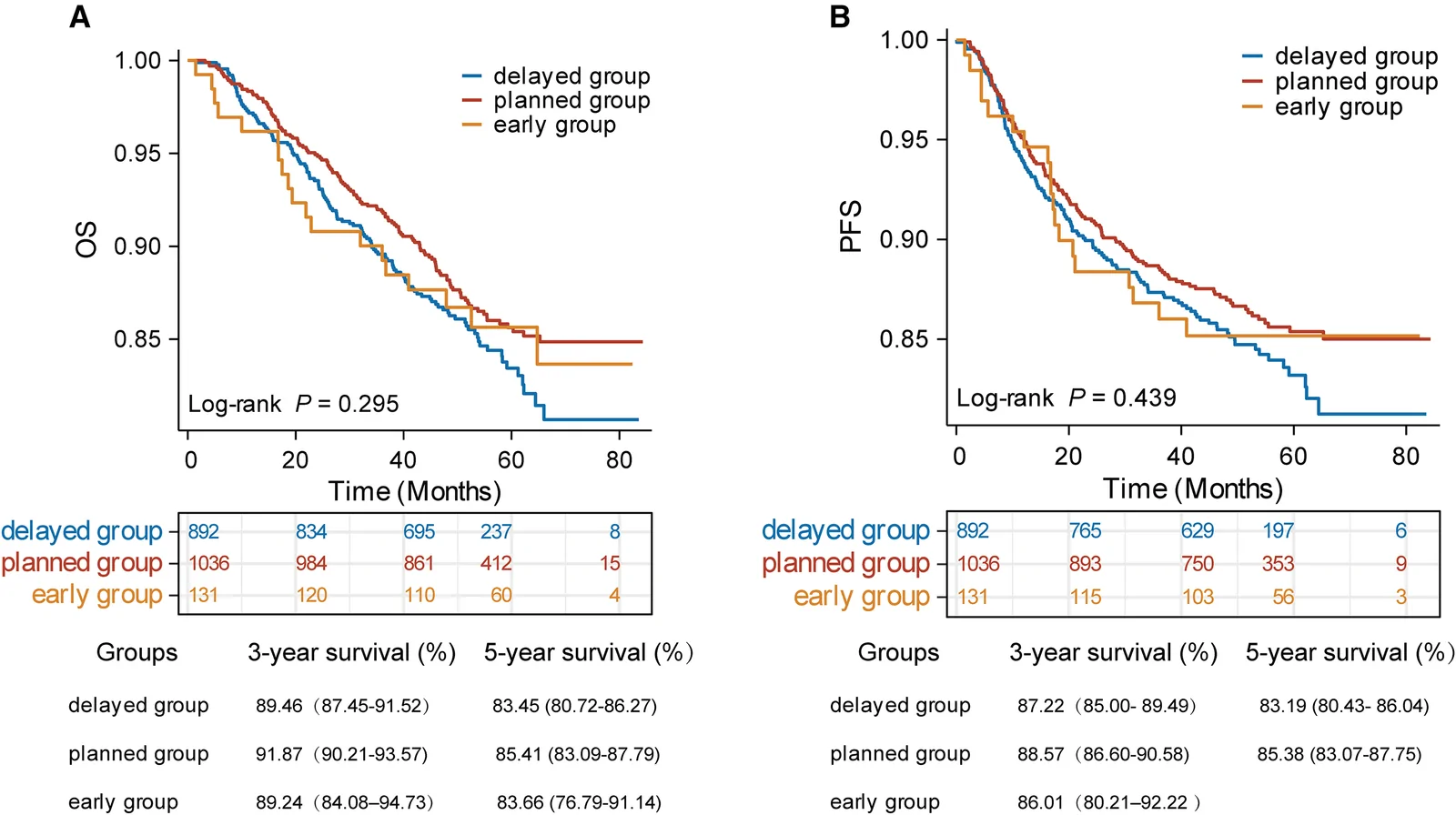
Survival outcomes of patients with NPC stratified by RTT completion status Comparison of (A) OS and (B) PFS probability between the planned completion group, the early completion group, and the delayed completion group. Abbreviations: Delayed group: the delayed completion group; planned group: the planned completion group; early group: the early completion group; OS, overall survival; PFS, progression-free survival.

The cohort was divided into three groups based on the timely completion of RT: (1) the planned completion group (ΔRTT = 0 days), (2) the early completion group (ΔRTT < 0 days), and (3) the delayed completion group (ΔRTT > 0 days). Subsequent analysis revealed that there were no notable differences in the factors related to the host and treatment among the three groups, with the exception of overall stage, treatment modality, and use of IC (all *p* < 0.05; Table 2). The planned completion group had a significantly higher proportion of patients receiving the IC + CCRT regimen (*p* < 0.001) and those with advanced overall stage disease (*p* < 0.05) compared to the other groups (Table 2). Although Kaplan-Meier curves for OS, PFS, LRFFS, and DMFFS did not show statistical significance among the ΔRTT groups (Figure 3; Figure S3), the planned completion group demonstrated numerically higher cumulative survival rates at equivalent time points compared to other groups across all four endpoints.

**Table 2:** Baseline patient characteristics according to ΔRTT

	No. (%) of patients by △RTT status			Total	P
	the planned completion group (*n* = 1036)	the early completion group (*n* = 131)	the delayed completion group (*n* = 892)	(*N* = 2059)	
Gender	–	–	–	–	0.51
Male	758(73.17)	101(77.10)	645(72.31)	1504(73.05)	–
Female	278(26.83)	30(22.90)	247(27.69)	555(26.95)	–
Age, year	–	–	–	–	0.795
≤45	392(37.84)	46(35.11)	329(36.88)	767(37.25)	–
>45	644(62.16)	85(64.89)	563(63.12)	1292(62.75)	–
T stage (8th edition)	–	–	–	–	0.13
T1	174(16.80)	20(15.27)	184(20.63)	378(18.36)	–
T2	169(16.31)	25(19.08)	131(14.69)	325(15.78)	–
T3	461(44.50)	51(38.93)	361(40.47)	873(42.40)	–
T4	232(22.39)	35(26.72)	216(24.22)	483(23.46)	–
N stage (8th edition)	–	–	–	–	0.856
N0	81(7.82)	9(6.87)	71(7.96)	161(7.82)	–
N1	382(36.87)	55(41.98)	342(38.34)	779(37.83)	–
N2	373(36.00)	44(33.59)	298(33.40)	715(34.73)	–
N3	200(19.31)	23(17.56)	181(20.29)	404(19.62)	–
Overall stage (8th edition)	–	–	–	–	< **0.05**
I	22(2.12)	1(0.76)	28(3.14)	51(2.48)	–
II	105(10.14)	24(18.32)	93(10.43)	222(10.78)	–
III	496(47.88)	52(39.69)	390(43.72)	938(45.56)	–
IV	413(39.86)	54(41.22)	381(42.71)	848(41.19)	–
EBV DNA titer, copies	–	–	–	–	0.878
negative	736(71.04)	94(71.76)	643(72.09)	1473(71.54)	–
positive	300(28.96)	37(28.24)	249(27.91)	586(28.46)	–
HGB, g/L	–	–	–	–	0.919
<113	271(26.16)	34(25.95)	223(25.00)	528(25.64)	–
113–151	723(69.79)	90(68.70)	628(70.40)	1441(69.99)	–
>151	42(4.05)	7(5.34)	41(4.60)	90(4.37)	–
Prescribed fractions	–	–	–	–	0.422
28–31	143(13.80)	20(15.27)	142(15.92)	305(14.81)	–
32–36	893(86.20)	111(84.73)	750(84.08)	1754(85.19)	–
Treatment modality	–	–	–	–	< **0.001**
RT alone	54(5.21)	6(4.58)	45(5.04)	105(5.10)	–
IC + RT	201(19.40)	30(22.90)	262(29.37)	493(23.94)	–
IC + RT + AC	23(2.22)	7(5.34)	22(2.47)	52(2.53)	–
CCRT	54(5.21)	14(10.69)	73(8.18)	141(6.85)	–
CCRT+AC	13(1.25)	1(0.76)	11(1.23)	25(1.21)	–
IC + CCRT	612(59.07)	65(49.62)	429(48.09)	1106(53.72)	–
IC+CCRT+AC	79(7.63)	8(6.11)	50(5.61)	137(6.65)	–
Residual or not	–	–	–	–	0.931
No	840(81.08)	108(82.44)	725(81.28)	1673(81.25)	–
Yes	196(18.92)	23(17.56)	167(18.72)	386(18.75)	–
NLR	–	–	–	–	0.455
Normal	540(52.12)	61(46.56)	452(50.67)	1053(51.14)	–
Abnormal	496(47.88)	70(53.43)	440(49.33)	1006(48.86)	–
Number of IC sessions	–	–	–	–	< **0.05**
0	121(11.68)	21(16.03)	129(14.46)	271(13.16)	–
≤3	563(54.34)	60(45.80)	428(47.98)	1051(51.04)	–
>3	352(33.98)	50(38.17)	335(37.56)	737(35.79)	–

RTT, radiotherapy treatment time; ΔRTT: the deviation in RTT; EBV-DNA, plasma Epstein-Barr virus (EBV) DNA; HGB, hemoglobin; RT, radiotherapy; IC + RT, radiotherapy following induction chemotherapy; CCRT, concurrent chemoradiotherapy; IC + CCRT, induction chemotherapy plus concurrent chemoradiotherapy; NLR, neutrophil-to-lymphocyte ratio; AC, adjuvant chemotherapy; IC, induction chemotherapy. ∗ Percentages may not add up to 100 due to rounding.

**Figure 4:**
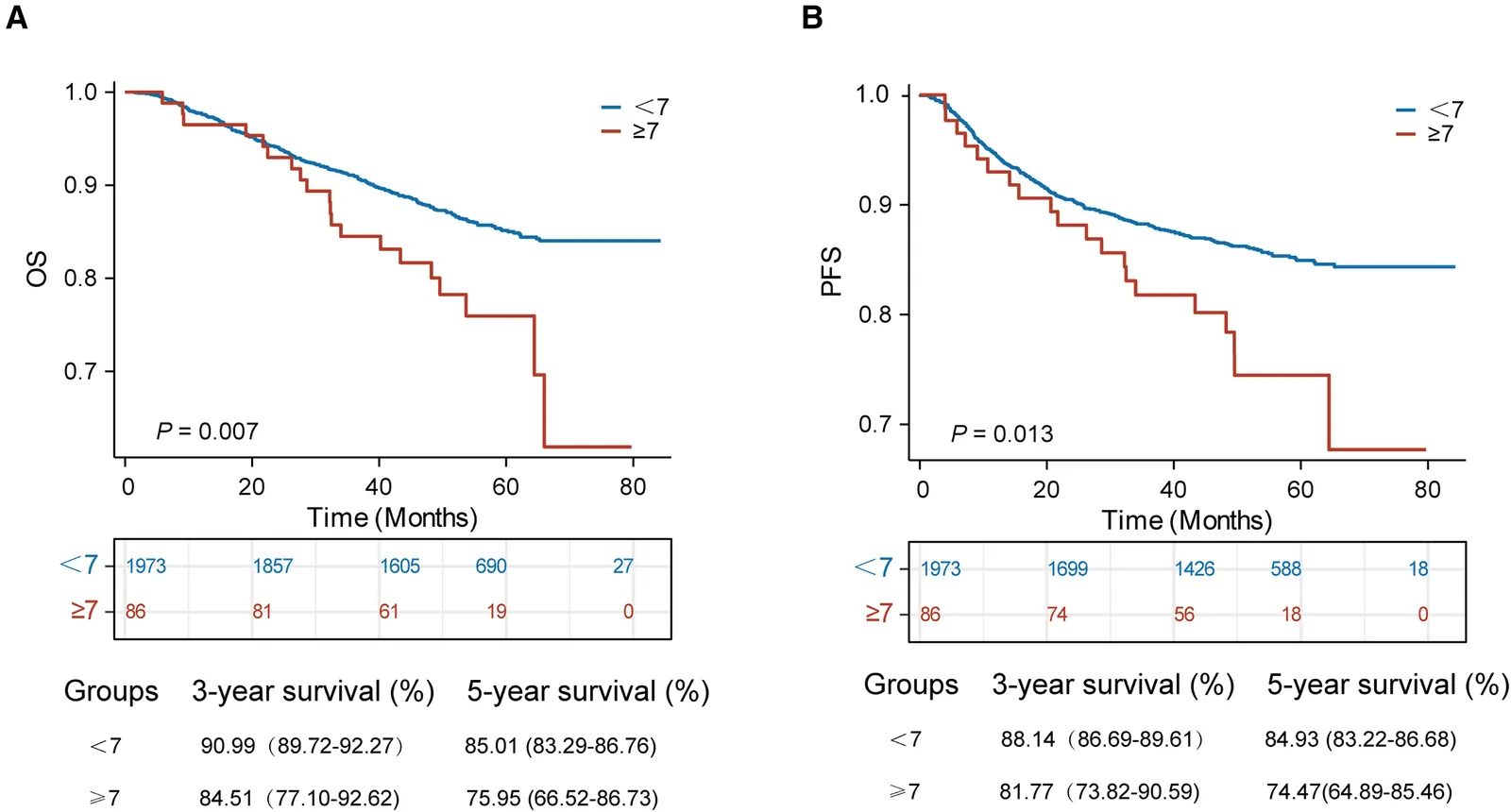
Kaplan-Meier survival curves for ΔRTT stratified by 7 days (A) OS, (B) PFS. Abbreviations: RTT, radiotherapy treatment time; ΔRTT, the deviation in RTT; OS, overall survival; PFS, progression-free survival.

Consequently, RPA was utilized to determine the optimal ΔRTT threshold for predicting substantial differences in survival rates. The optimal cutoff for OS-related ΔRTT was determined to be 7 days. Accordingly, the cohort was divided into high ΔRTT (≥7 days) and low ΔRTT (<7 days) groups for survival analysis. Kaplan-Meier curves demonstrated that patients with ΔRTT ≥ 7 days had worse outcomes in OS, PFS, LRFFS, and DMFFS compared to those with ΔRTT<7 days (*p* < 0.05, Figure 4; Figure S4). Multivariate analysis, accounting for various prognostic factors, confirmed ΔRTT as an independent adverse prognostic factor for OS, PFS, LRFFS, and DMFFS (all *p* < 0.05; Tables 3 and 4; Tables S3 and S4).

**Table 3:** Univariate and multivariate analyses of overall survival

Characteristic	Univariate analysis		Multivariate analysis	
	HR (95% CI)	P	HR (95% CI)	P
**Gender**				
Female	Ref.	–	Ref.	–
Male	1.534 (1.148–2.050)	**0.004**	1.450 (1.077–1.951)	**0.014**
**Age, year**				
>45	Ref.	–	Ref.	–
≤45	0.591 (0.456–0.767)	< **0.001**	0.614 (0.470–0.802)	< **0.001**
**Overall stage (8th edition)**				
I	Ref.	–	Ref.	–
II	1.376 (0.308–6.146)	0.676	2.072 (0.419–10.255)	0.372
III	2.475 (0.609–10.056)	0.205	7.980 (1.783–35.722)	**0.007**
IV	6.530 (1.621–26.302)	**0.008**	19.915 (4.423–89.670)	< **0.001**
**EBV DNA titer, copies**				
negative	Ref.	–	Ref.	–
positive	2.129 (1.687–2.686)	< **0.001**	1.906 (1.492–2.434)	< **0.001**
**HGB, g/L**				
<113	Ref.	–	Ref.	–
113-151	0.866 (0.669–1.121)	0.275	0.869 (0.661–1.142)	0.313
>151	0.318 (0.129–0.785)	**0.013**	0.555 (0.211–1.460)	0.233
**Prescribed fractions**				
28-31	Ref.	–	Ref.	–
32-36	1.668 (1.136–2.448)	**0.009**	1.024 (0.679–1.546)	0.909
**Treatment modality**				
RT alone	Ref.	–	Ref.	–
IC + RT	0.845 (0.511–1.399)	0.513	0.265 (0.149–0.473)	< **0.001**
IC + RT + AC	1.506 (0.744–3.049)	0.255	0.363 (0.169–0.783)	**0.010**
CCRT	0.438 (0.213–0.903)	**0.025**	0.552 (0.250–1.222)	0.143
CCRT+AC	0.414 (0.097–1.779)	0.236	0.801 (0.168–3.832)	0.781
IC + CCRT	0.692 (0.429–1.118)	0.133	0.224 (0.130–0.385)	< **0.001**
IC+CCRT+AC	1.078 (0.602–1.931)	0.800	0.308 (0.163–0.584)	< **0.001**
**Residual or not**				
Yes	Ref.	–	–	–
No	0.977 (0.729–1.308)	0.874	–	–
**NLR**				
Abnormal	Ref.	–	–	–
Normal	0.777 (0.616–0.979)	**0.033**	–	–
**Number of IC sessions**				
0	Ref.	–	–	–
≤3	1.192 (0.817–1.740)	0.363	–	–
>3	1.394 (0.944–2.057)	0.095	–	–
**ΔRTT, days**				
<7	Ref.	–	Ref.	–
≥7	1.842 (1.169–2.902)	**0.008**	1.879 (1.189–2.969)	**0.007**
**Interruption times**				
0	Ref.	–	–	–
1	0.894 (0.681–1.172)	0.417	–	–
>1	1.165 (0.864–1.572)	0.316	–	–

RTT, radiotherapy treatment time; ΔRTT: the deviation in RTT; EBV-DNA, plasma Epstein-Barr virus (EBV) DNA; HGB, hemoglobin; RT, radiotherapy; IC + RT, radiotherapy following induction chemotherapy; CCRT, concurrent chemoradiotherapy; IC + CCRT, induction chemotherapy plus concurrent chemoradiotherapy; NLR, neutrophil-to-lymphocyte ratio; AC, adjuvant chemotherapy; IC, induction chemotherapy.

**Table 4:** Univariate and multivariate analyses of progression-free survival

Characteristic	Univariate analysis		Multivariate analysis	
	HR (95% CI)	P	HR (95% CI)	P
**Gender**				
Female	Ref.	–	Ref.	–
Male	1.554 (1.163–2.077)	**0.003**	1.449 (1.076–1.951)	**0.015**
**Age, year**				
>45	Ref.	–	Ref.	–
≤45	0.621 (0.479–0.806)	< **0.001**	0.658 (0.504–0.859)	**0.002**
**Overall stage (8th edition)**				
I	Ref.	–	Ref.	–
II	1.385 (0.310–6.187)	0.670	1.997 (0.405–9.852)	0.396
III	2.526 (0.622–10.265)	0.195	7.147 (1.597–31.971)	**0.010**
IV	6.820 (1.693–27.471)	**0.007**	18.075 (4.018–81.301)	< **0.001**
**EBV DNA titer, copies**				
negative	Ref.	–	Ref.	–
positive	2.231 (1.768–2.815)	< **0.001**	1.988 (1.556–2.540)	< **0.001**
**HGB, g/L**				
<113	Ref.	–	Ref.	–
113-151	0.899 (0.695–1.163)	0.417	0.891 (0.678–1.171)	0.409
>151	0.321 (0.130–0.792)	**0.014**	0.555 (0.211–1.459)	0.233
**Prescribed fractions**				
28-31	Ref.	–	Ref.	–
32-36	1.688 (1.150–2.479)	**0.007**	1.003 (0.664–1.514)	0.990
**Treatment modality**				
RT alone	Ref.	–	Ref.	–
IC + RT	0.877 (0.530–1.452)	0.610	0.299 (0.167–0.532)	< **0.001**
IC + RT + AC	1.650 (0.815–3.341)	0.164	0.428 (0.198–0.923)	**0.031**
CCRT	0.452 (0.219–0.930)	**0.031**	0.571 (0.260–1.258)	0.165
CCRT+AC	0.421 (0.098–1.809)	0.245	0.811 (0.170–3.875)	0.793
IC + CCRT	0.732 (0.453–1.182)	0.202	0.261 (0.152–0.449)	< **0.001**
IC+CCRT+AC	1.246 (0.696–2.232)	0.459	0.385 (0.204–0.728)	**0.003**
**Residual or not**				
Yes	Ref.	–	–	–
No	0.943 (0.705–1.263)	0.695	–	–
**NLR**				
Abnormal	Ref.	–	Ref.	–
Normal	0.778 (0.617–0.982)	**0.034**	0.820 (0.649–1.036)	0.096
**Number of IC sessions**				
0	Ref.	–	–	–
≤3	1.256 (0.861–1.833)	0.237	–	–
>3	1.427 (0.967–2.106)	0.073	–	–
**ΔRTT, days**				
<7	Ref.	–	Ref.	–
≥7	1.767 (1.122–2.783)	**0.014**	1.791 (1.133–2.831)	**0.013**
**Interruption times**				
1	Ref.	–	–	–
0	1.141 (0.869–1.497)	0.343	–	–
>1	1.285 (0.921–1.791)	0.140	–	–

RTT, radiotherapy treatment time; ΔRTT: the deviation in RTT; EBV-DNA, plasma Epstein-Barr virus (EBV) DNA; HGB, hemoglobin; RT, radiotherapy; IC + RT, radiotherapy following induction chemotherapy; CCRT, concurrent chemoradiotherapy; IC + CCRT, induction chemotherapy plus concurrent chemoradiotherapy; NLR, neutrophil-to-lymphocyte ratio; AC, adjuvant chemotherapy; IC, induction chemotherapy.

### Univariate and multivariate analysis

Univariate analysis, detailed in Tables 3 and 4 and Tables S3 and S4, revealed significant differences in OS, PFS, LRFFS, and DMFFS based on factors such as age, sex, overall stage, EBV-DNA (pre-treatment EBV-DNA) levels, HGB (pre-treatment HGB) levels, radiation fractions, treatment modality, NLR, and ΔRTT (*p* < 0.05). The interruption count reflects the number of treatment suspensions during RT, with 0 times indicating uninterrupted treatment completion, 1 time representing a single interruption event during the course, and >1 times denoting multiple interruptions. Multivariate analysis identified sex, age, stage, treatment modality, EBV-DNA levels, and ΔRTT as independent prognostic variables for OS, PFS, LRFFS, and DMFFS (*p* < 0.05, Tables 3 and 4; Tables S3 and S4). According to the RPA results, the grouping criteria for ΔRTT were defined as: ΔRTT < 7 days vs. ≥7 days. Patients with ΔRTT ≥ 7 days had a 1.879-fold higher risk of mortality (95% CI 1.189–2.969, *p* = 0.007) and a 1.791-fold higher risk of disease progression (95% CI 1.133–2.831, *p* = 0.013) compared to those with ΔRTT < 7 days.

### Interaction of ΔRTT with other covariates

Based on RPA results, patients were stratified into ΔRTT < 7 days and ≥ 7 days groups. As shown in Figure S5, the results demonstrate a significant interaction between ΔRTT and interruption frequency. Subgroup analyses demonstrated significantly improved OS in low-risk versus high-risk patients among females, those aged > 45 years, receiving 28–31 RT fractions, with normal NLR levels, undergoing zero cycles of IC, and experiencing a single treatment interruption (Figure S5).

### Analysis of survival effects of continuous interruption

**Figure 5:**
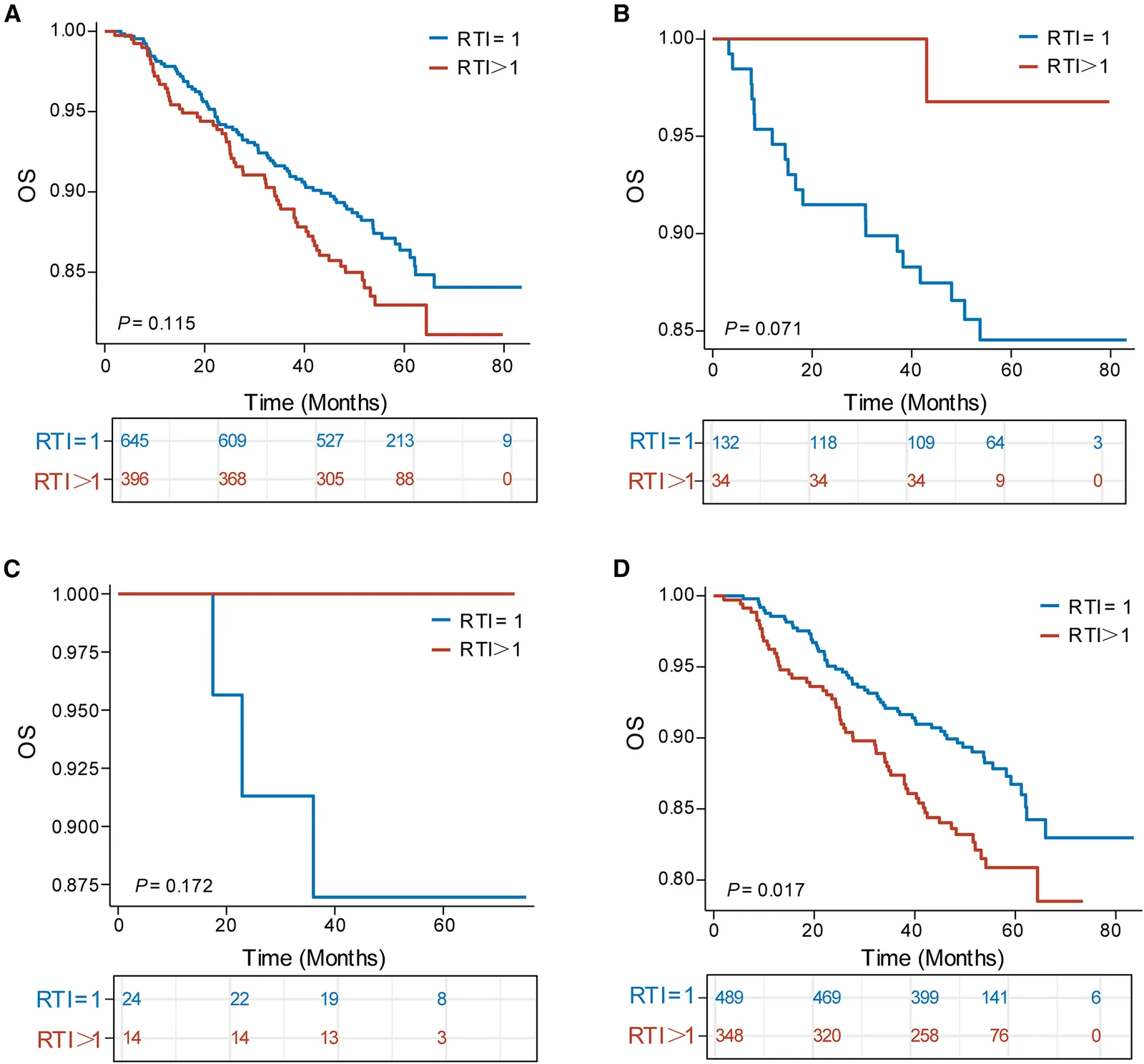
Kaplan-Meier curves for different populations according to the number of radiotherapy interruptions (interruptions of 1 and more than 1) (A) Total population. (B) Population with ΔRTT = 0 days. (C) Population with ΔRTT <0 days. (D) Population with ΔRTT >0 days. Abbreviations: RTT, radiotherapy treatment time; ΔRTT, the deviation in RTT.

**Figure 6:**
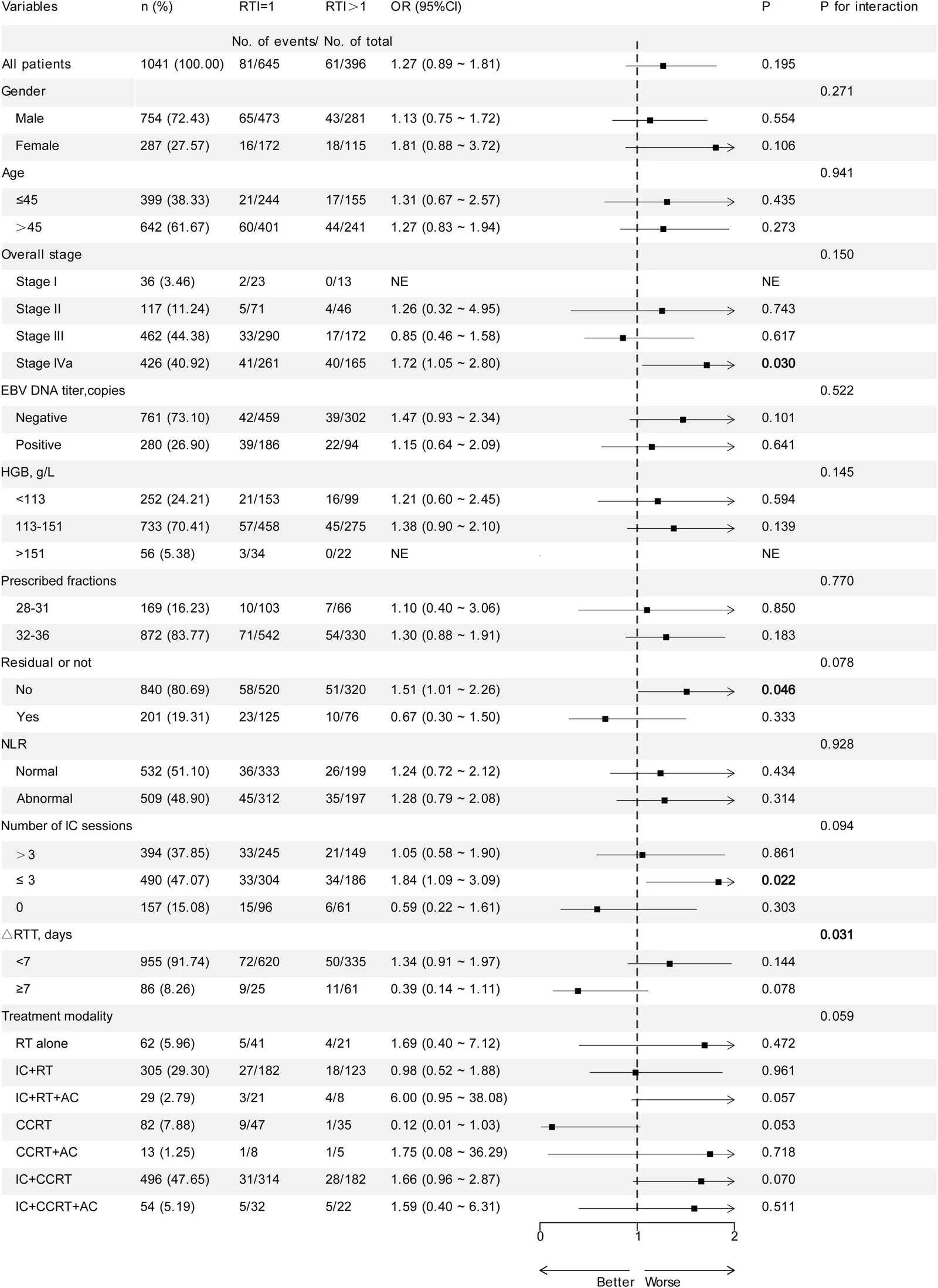
Subgroup analysis according to number of radiotherapy interruptions (1 and more than1interruption) NE, not estimable because of zero events or complete separation.

Patients were stratified into two cohorts based on the frequency of treatment interruptions: a single interruption group and a multiple interruptions group. An analysis of Kaplan-Meier curves indicated that the OS of patients with ΔRTT > 0 exhibited a statistically significant difference (*p* < 0.05; Figure 5). Subsequent subgroup analyses were conducted to evaluate the impact of the number of interruptions across various parameters. The results shown in Figure 6 demonstrate a significant interaction between ΔRTT and interruption frequency. The correlation between the number of interruptions and OS remained consistent across stage IV, patients receiving IC ≤ 3, and those without residual focus (*p* < 0.05, Figure 6).

## Discussion

Our study revealed that an extension of seven days or more in the ΔRTT for patients with NPC correlates with a poorer prognosis. Furthermore, our findings suggest that the frequency of RTIs may influence survival outcomes in these patients, particularly when RT is prolonged.

In the era of IMRT, significant debate persists about the impact of interrupted RT on patients with tumor. Some evidence suggests that interruptions do not significantly affect the prognosis of patients with head and neck tumors and anorectal squamous cell carcinoma.^20^^,^^21^ However, Xu et al. demonstrated that interruptions in RT yield varied prognostic outcomes in NPC,^12^ highlighting the lack of consensus regarding the prognostic implications of different modes of RTI.

In a retrospective study conducted by Li et al., no statistically significant correlation was identified between survival outcomes and the duration of RTT within the interval of 36–63 days.^22^ Conversely, our research indicated that prolonged RTT is associated with diminished survival outcomes. Further analysis revealed that an RTT of 50 days or more particularly correlates with poorer prognostic outcomes. However, the variability in TNM stages among patients, implying differences in RT session frequency, challenges the accuracy of prognosis assessment for patients undergoing fewer RT fractions within this 50-day cut-off period.

Therefore, we conducted an analysis to evaluate the impact of RTT on patient survival, focusing on adherence to the RT schedule. Contrary to expectations, although OS and PFS rates were higher among patients who completed their RT on schedule compared to those with earlier or extended completion times, the higher proportion of patients with advanced TNM staging within the on-schedule group—known to be associated with poorer survival outcomes—may have influenced these results. Consequently, there was no statistical significance among the survival curves (*p* > 0.05). To ensure the study’s generalizability across patients at various stages and with diverse treatment options, we further investigated the impact of ΔRTT on survival outcomes.

The prognostic relevance of ΔRTT remains controversial, as evidenced by several previous retrospective studies. Kong et al. reported that a median interruption of three days was associated with a significantly poorer prognosis (*p* = 0.046).^23^ Xu et al. demonstrated that a four-day interruption threshold significantly affected both PFS and OS.^24^ Shyh-An Yeh et al. determined that interruptions of five days or more during IMRT, irrespective of concurrent chemotherapy, significantly compromise OS in patients with NPC.^25^ The observed variations in ΔRTT across these studies may be attributable to limited sample sizes, brief follow-up durations, or the analysis of ΔRTT as a categorical variable. To obtain a more precise measure, we examined a cohort of 2,059 patients with extended follow-up periods and conducted a continuous analysis of ΔRTT. Our findings corroborated those of Yao et al., emphasizing the necessity of limiting RTIs to fewer than seven days to mitigate the risk of adverse health outcomes.^26^

We also examined the correlation between interruptions in continuous or decentralized RT and the prognosis of patients with NPC. In our univariate and multivariate analyses, continuous interruptions in RT did not emerge as an independent adverse prognostic factor for the four survival endpoints. However, when patients were stratified based on the frequency of RTIs (one or more times), Kaplan-Meier survival analysis revealed that OS, PFS, LRFFS, and DMFFS rates were lower in patients experiencing more than one interruption compared to those with a single interruption. Nevertheless, statistical significance was only observed in the Kaplan-Meier curve for the group with a ΔRTT greater than zero. These findings suggest that patients who exceed the prescribed duration of RT should minimize the number of interruptions to optimize treatment outcomes. The adverse impact of prolonged RTT and multiple interruptions observed in our cohort is fundamentally driven by accelerated repopulation. This radiobiological phenomenon, characterized by the rapid proliferation of tumor cells during treatment gaps, serves as the biological basis for maintaining RT continuity. During these delays, surviving clonogens accelerate their growth, effectively diminishing the biologically effective dose (BED) as subsequent fractions are partially consumed to counteract new cell production. Within the framework of the “Four Rs” of radiobiology, such interruptions allow the tumor to outpace cumulative radiation damage, thereby compromising local control and survival. Therefore, minimizing treatment disruptions is critical to preventing this proliferative recovery and ensuring optimal therapeutic efficacy in NPC.

In this study, databases pertaining to RTT and ΔRTT were developed using data from 2,059 patients who experienced interruptions in their RT for various reasons. By assessing multiple survival endpoints, including OS, PFS, LRFFS, and DMFFS, it was determined that a ΔRTT of seven days or more may lead to poor prognosis in patients with NPC. This finding aligns with results from a large cohort study conducted at another center, further substantiating that a ΔRTT of seven days or more may be a critical factor influencing the efficacy of RT in these patients. Additionally, the study elucidates the differential impact of ΔRTT across various patient subgroups, categorized by overall stage and chemotherapy modalities, thereby providing insights that can inform personalized treatment strategies. Furthermore, this study demonstrates that the number of RTIs influences survival outcomes in patients with NPC who exceed the prescribed duration of treatment. This finding sheds light on a previously unexamined aspect of how continuous versus dispersed RTIs impact patient prognosis. In clinical practice, various dose-compensation protocols are employed to mitigate the effects of RTIs. Mainstream strategies typically involve increasing the dose per fraction or utilizing twice-daily treatments to maintain the BED when the overall treatment time is extended. At our institution, we follow a similar protocol where interruptions longer than 3 days require dose compensation through fraction size adjustments. However, our results demonstrate that a ΔRTT of 7 days or more still significantly correlates with a poorer prognosis despite these interventions. This underscores the limitation of current compensation methods in completely counteracting accelerated repopulation, particularly when multiple interruptions occur. For clinical oncologists, this highlights the necessity of prioritizing treatment continuity and keeping ΔRTT below the 7-day threshold even when compensation protocols are available.

Our study indicates that a ΔRTT of seven days or more constitutes a critical factor contributing to poor prognosis in patients with NPC undergoing IMRT. Additionally, recurrent interruptions in RT are associated with adverse prognostic outcomes. Consequently, it is imperative in clinical practice to enhance RT management protocols to ensure that ΔRTT remains below seven days and to minimize the frequency of RTIs.

### Limitations of the study

Despite the strengths of the study, several limitations must be acknowledged. Being retrospective, the study is susceptible to inherent biases and confounding factors that could have influenced the results. Specifically, due to the retrospective nature of our database, we were unable to granularly distinguish between the etiologies of interruptions, such as treatment-related toxicities versus non-medical factors (e.g., public holidays or machine maintenance). While these different causes might theoretically reflect different aspects of patient frailty or radiobiological impact, our findings prioritize the cumulative ΔRTT as a pragmatically critical determinant of survival. Additionally, the research was conducted at a single institution, potentially limiting the generalizability of the findings. Future research should prioritize prospective, multicenter studies to validate these results and further investigate the biological mechanisms underlying the impact of RTIs on the prognosis of NPC.

## Resource availability

### Lead contact

Requests for further information and resources should be directed to and will be fulfilled by the lead contact, Fengjie Lin (6619941@qq.com).

### Materials availability

This study did not generate new unique reagents.

### Data and code availability

• Data reported in this paper will be shared by the lead contact upon request.
• This study did not generate any original code.
• Any additional information required to reanalyze the data reported in this paper is available from the lead contact upon request.

## Acknowledgments

This work was supported by the grants of the 10.13039/501100001809National Natural Science Foundation of China of China (82473376); the Joint Funds for the Innovation of Science and Technology, Fujian province (2025Y9674, 2025Y9665); the Major Scientific Research Program for Young and Middle-aged Health Professionals of Fujian Province, China (2021ZQNZD010); Science and Technology Pilot Program of Fujian Province, China (2025Y0043); the Natural Science Foundation of Fujian Province (2024J011086), and High-level Talent Training Program of Fujian Cancer Hospital (2022YNG07), Subsidy for Young and Middle-aged Experts with Outstanding Contributions in Fujian Province's Health System in 2021–2022 (F23R-TG01-01); Fujian Clinical Research Center for Radiation and Therapy of Digestive, Respiratory and Genitourinary Malignancies (2021Y2014).

## Author contributions

W. X.W., C.L., and Z.W.H. designed this study. W.X.W., C.L., Z.W.H., Y.L., X.Y.H. and X.Y.L. contributed to the data collection. W.X.W., Y.X.Y. and P.D.O. analyzed the data. S.F.Q. supervised the study. W.X.W.,Z.W.H., and F.J.L. wrote the manuscript. All authors read and approved the final manuscript.

## Declaration of interests

The authors declare no competing interests.

## STAR★Methods

### Key resources table

**Table:** 

REAGENT or RESOURCE	SOURCE	IDENTIFIER
**Software and algorithms**		

R (version 3.3.1)	The R Foundation	https://www.r-project.org/

### Experimental model and study participant details

#### Patients

This retrospective study involved a cohort of patients treated with IMRT at Fujian Cancer Hospital between January 2017 and December 2019. All participants were newly diagnosed with non-metastatic NPC, confirmed pathologically, and had complete clinical data. The patients underwent IMRT as planned, with or without CCRT, induction chemotherapy (IC), and adjuvant chemotherapy (AC). Daily RT data were accessed through the MOSAIQ integrated platform. Ultimately, 2,059 patients were enrolled in the study.

#### Ethics approval and security

The study received ethical approval from the Fujian Cancer Hospital's Research Ethics Committee (Fujian, China) and adhered to the principles of the Declaration of Helsinki (K2024-517-01). Given the retrospective nature of the research, the requirement for informed consent was waived. All procedures were performed in accordance with the relevant guidelines and regulations. For the cohort of NPC patients, we compiled comprehensive clinical and demographic profiles including age, TNM stage, and other tumor-related characteristics. Complete sample distributions and detailed baseline characteristics of this study population are provided in Tables 1 and 2.

#### Influence of sex on the results

Among the 2,059 patients enrolled, 1,504 (73.05%) were male and 555 (26.95%) were female. Both univariate and multivariate analyses identified sex as a significant independent prognostic factor for OS, PFS, LRFFS, and DMFFS (*p* < 0.05, Tables 3 and 4; Tables S3 and S4). Additionally, subgroup analysis revealed that the survival benefit of maintaining a low ΔRTT was particularly pronounced and statistically significant in female patients.

### Method details

#### Treatment and evaluation

Radical IMRT was administered using simultaneous integrated boost with 6MV photons. All patients were immobilized in a supine position utilizing a thermoplastic mask. The delineation of target volumes and identification of organs at risk were facilitated by the fusion of computed tomography (CT)/magnetic resonance imaging (MRI) images. The gross tumor volume (GTV) included all visible disease, comprising nasopharyngeal primary tumors (GTV-T) and positive lymph nodes (GTV-N), as identified through imaging, clinical examination, and endoscopic findings. The clinical target volume (CTV) was defined as the GTV along with its surrounding subclinical lesions. A planned target volume (PTV) was established as a safety margin around the GTV/CTV, incorporating an additional 3mm to account for positional uncertainties and intrinsic organ movements. Radiation doses prescribed for NPC adhered to our institution's guidelines, specifying a dose range of 6600 to 7310 cGy for PTV-GTV-T, 6000-7310 cGy for PTV-GTV-N, and 5000 to 6600 cGy for PTV-CTV across 28 to 36 fractions delivered once daily, five times per week. Dose constraints for organs at risk were determined in accordance with the RTOG 0225 protocol. For unscheduled treatment interruptions, our institutional protocol required immediate resumption of the original dose/fractionation schedule for interruptions ≤3 days, with replanning required for longer delays, while dose compensation through fraction size adjustments maintained the total prescribed dose. Chemotherapy regimens were tailored to each patient based on overall stage, physical condition, drug availability, and comorbidities. Treatment modalities were categorized into seven groups: RT alone, IC+RT, IC+RT+AC, CCRT, CCRT+AC, IC+CCRT and IC+CCRT+AC. According to guidelines, IMRT is recommended for stage I NPC, while stages II to IVa are managed with platinum-based CCRT, with the option of adding IC or AC.

#### Follow-up and endpoints

Patients participated in systematic follow-up evaluations, which encompassed clinical examinations, nasopharyngoscopy, and imaging modalities such as CT, MRI, and positron emission tomography-CT. These assessments were conducted quarterly during the initial two-year period, biannually over the subsequent three years, and annually thereafter. The most recent follow-up was conducted on May 20, 2024. The principal aim of this study was to assess OS, which is delineated as the interval from the initiation of radiation therapy to the occurrence of death from any cause or the most recent verified date of survival. Secondary outcomes encompassed progression-free survival (PFS), locoregional failure-free survival (LRFFS), and distant metastasis failure-free survival (DMFFS). PFS was delineated as the interval commencing from the initiation of radiation therapy to the occurrence of disease progression, mortality, or the most recent follow-up, whichever event transpired first. LRFFS and DMFFS were defined as the duration from the start of radiation therapy to the first occurrence of local/regional recurrence, distant metastasis, mortality, or the most recent follow-up assessment. For patients who did not return for follow-up, information was obtained primarily via telephone, either from the patient, their family, or the medical records department.

#### The definitions of RTT and ΔRTT

Radiation treatment time (RTT) refers to the interval spanning from the commencement of RT to the conclusion of the prescribed therapeutic regimen. For patients undergoing IMRT, the deviation in RTT(ΔRTT) was determined by calculating the difference between the actual time required to complete the prescribed treatment regimen and the initially planned treatment duration. For example, if the prescribed dose was 70 Gy administered over 35 fractions, with a planned treatment duration of 47 days (commencing on a Monday) or 49 days (commencing on any weekday other than Monday), the ΔRTT was determined accordingly. Treatment interruptions were defined as missed days of scheduled radiotherapy occurring on workdays (Monday through Friday). While standard weekends were not considered interruptions, gaps resulting from public holidays falling on a weekday were included in the treatment interruptions count. In cases where a holiday fell on a weekend and the facility was closed on the preceding Friday, that Friday was also recorded as a treatment interruption.

### Quantification and statistical analysis

Covariates analyzed in this study included host factors (such as gender [male vs. female], age [≤45 years vs. >45 years], hemoglobin(HGB) levels, and neutrophil-to-lymphocyte ratio(NLR),^27^ tumor characteristics (including T stage, N stage, and overall stage), in addition to treatment-related variables (such as RTT, ΔRTT, the number of prescribed fractions, and the modality of treatment). Both HGB and NLR detections were performed respectively before treatment. Regarding NLR, it is calculated as the absolute neutrophil count divided by the absolute lymphocyte count. A normal NLR range is typically considered to be between 1 and 2. The concentration of plasma EBV-DNA was quantified using reverse transcription-quantitative PCR, with detectable vs. undetectable levels determined using a cut-off value of 0 copies/mL.^28^ Variables were categorized based on clinical findings, whereas continuous variables were transformed into categorical formats utilizing established cutoff points or results from previous studies. Clinicopathologic characteristics were compared across different groups using the chi-squared test or Fisher’s exact test for categorical variables. We conducted recursive partitioning analyses (RPAs) to determine the best RTT and ΔRTT thresholds. The recursive partitioning analysis (RPA) method determined optimal splitting nodes at each step to identify the most favorable RTT and ΔRTT thresholds through classification and regression tree-based analysis. Survival curves were estimated using the Kaplan-Meier method,^29^ with comparisons made using the log-rank test. Cox regression analysis was employed to calculate hazard ratios and 95% confidence intervals, quantifying the impact of potential confounders on survival outcomes. Additionally, a subgroup analysis was conducted to assess the impact of continuous interruptions. Statistical significance was defined as a *p*-value less than 0.05. R software, version 3.3.1(https://www.r-project.org/), was used for all statistical analyses.

The exact value of n, representing the total number of patients, along with comprehensive statistical details, is explicitly specified and can be found across the text of the results section, Tables 1, 2, 3, and 4, and the corresponding main and supplementary figure legends.
