# Supplementary material for: Prognostic value of radiation treatment time deviation in nasopharyngeal carcinoma receiving intensity-modulated radiotherapy
Source: iScience. 2026 Aug 13;29(9):117124. doi: 10.1016/j.isci.2026.117124 (PMC13499206; doi:10.1016/j.isci.2026.117124)
Supplement: Supplementary file 1 [file mmc1.pdf]

iScience, Volume *29*

Supplemental information

Prognostic value of radiation treatment time deviation in nasopharyngeal carcinoma receiving intensity-modulated radiotherapy

Wenxi Wu, Chao Li, Zongwei Huang, Ying Li, Xinyi Hong, Xiaoyong Liu, Yuxing Yu, Peidong Ou, Sufang Qiu, and Fengjie Lin

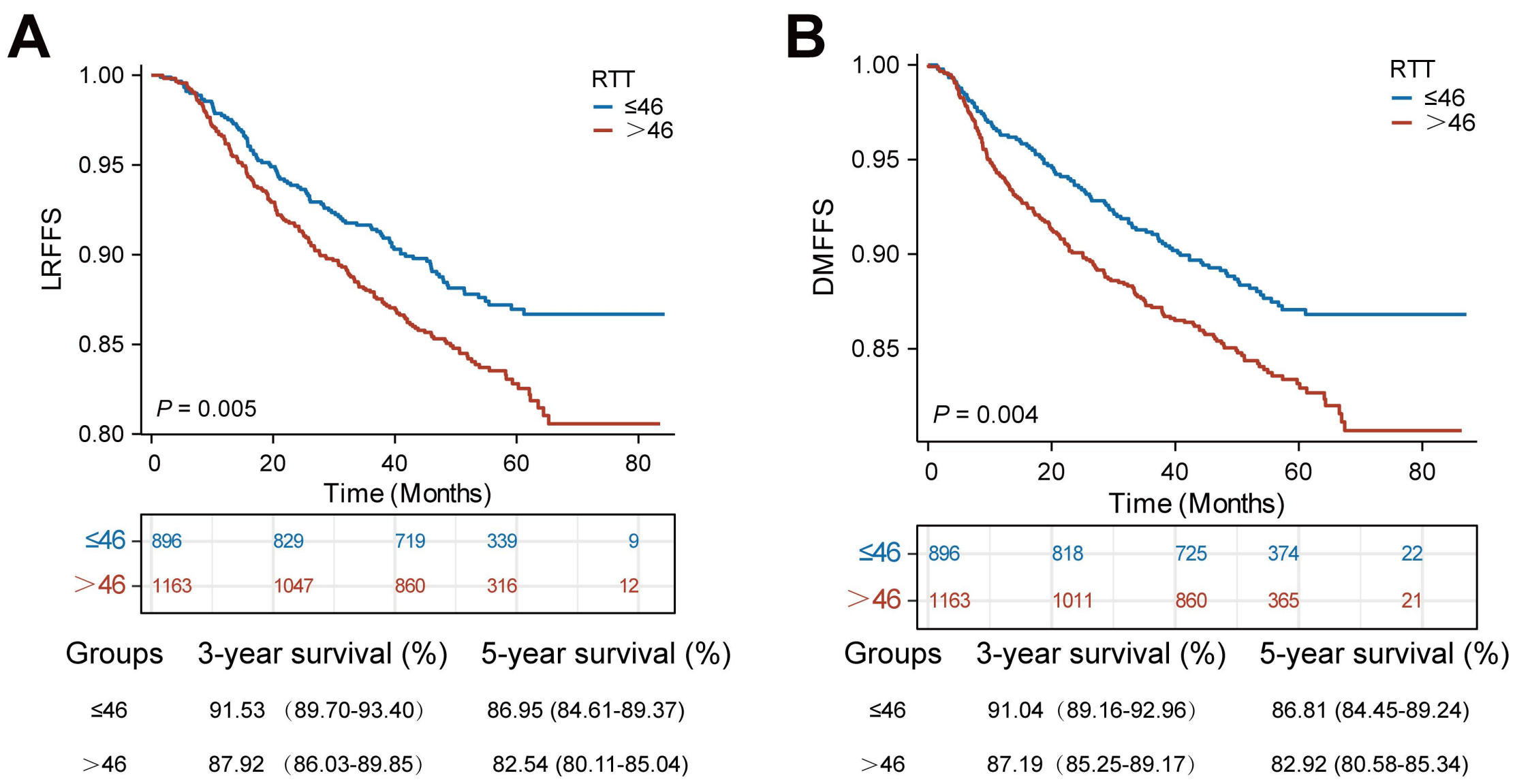

Supplementary Fig. 1. Kaplan–Meier's plots showed (A) LRFFS, (B) DMFFS divided by the RTT of ≤46 d, and >46 d, respectively. Abbreviations: RTT, radiotherapy treatment time; LRFFS, locoregional failure-free survival; DMFFS,distant metastasis failure-free survival.(Relates to Fig. 1)

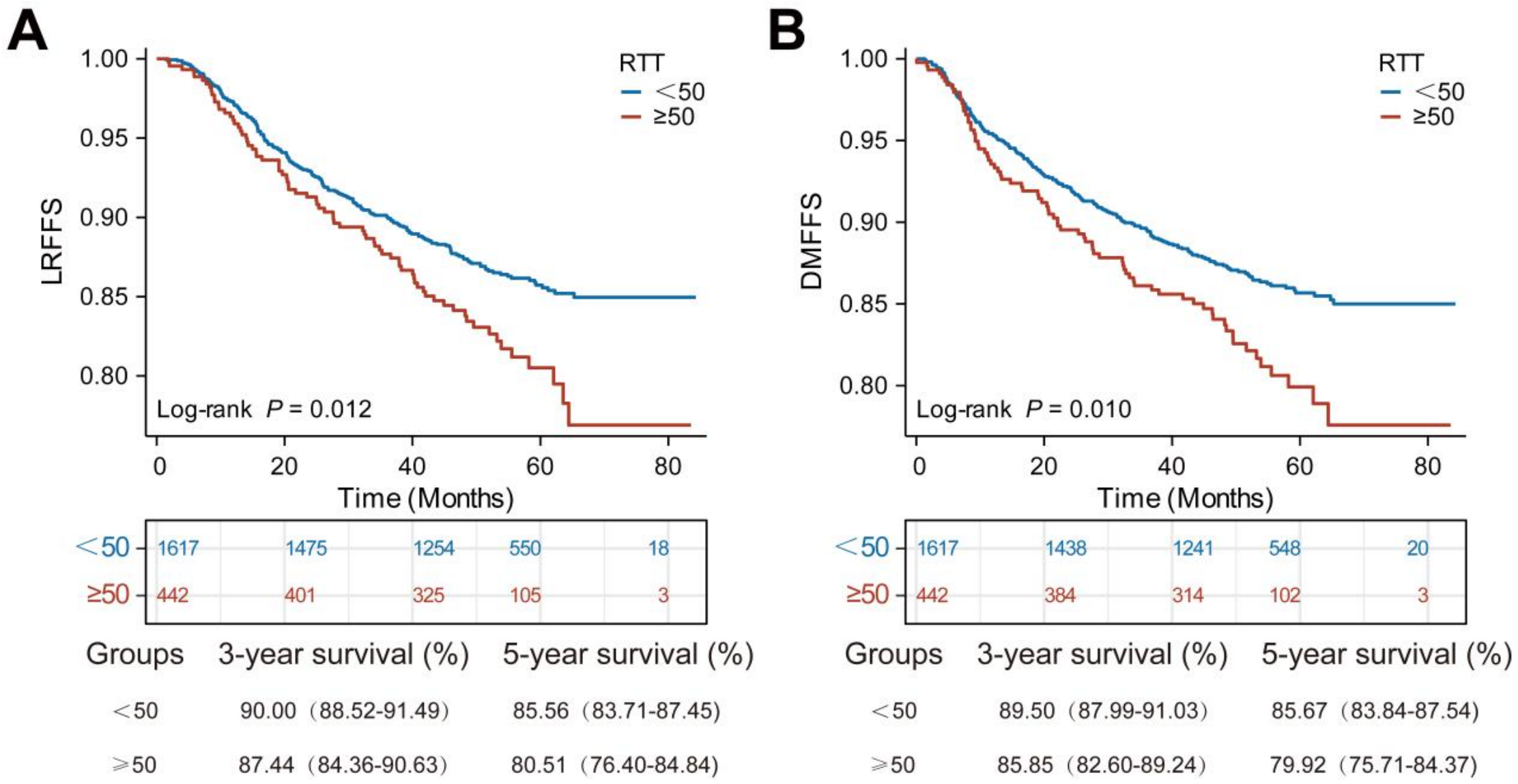

Supplementary Fig. 2. Kaplan–Meier survival curves illustrated (A) LRFFS, (B) DMFFS categorized by the RTT of <50 d and ≥50 d. Abbreviations: RTT, radiotherapy treatment time; LRFFS, locoregional failure-free survival; DMFFS,distant metastasis failure-free survival.(Relates to Fig. 2)

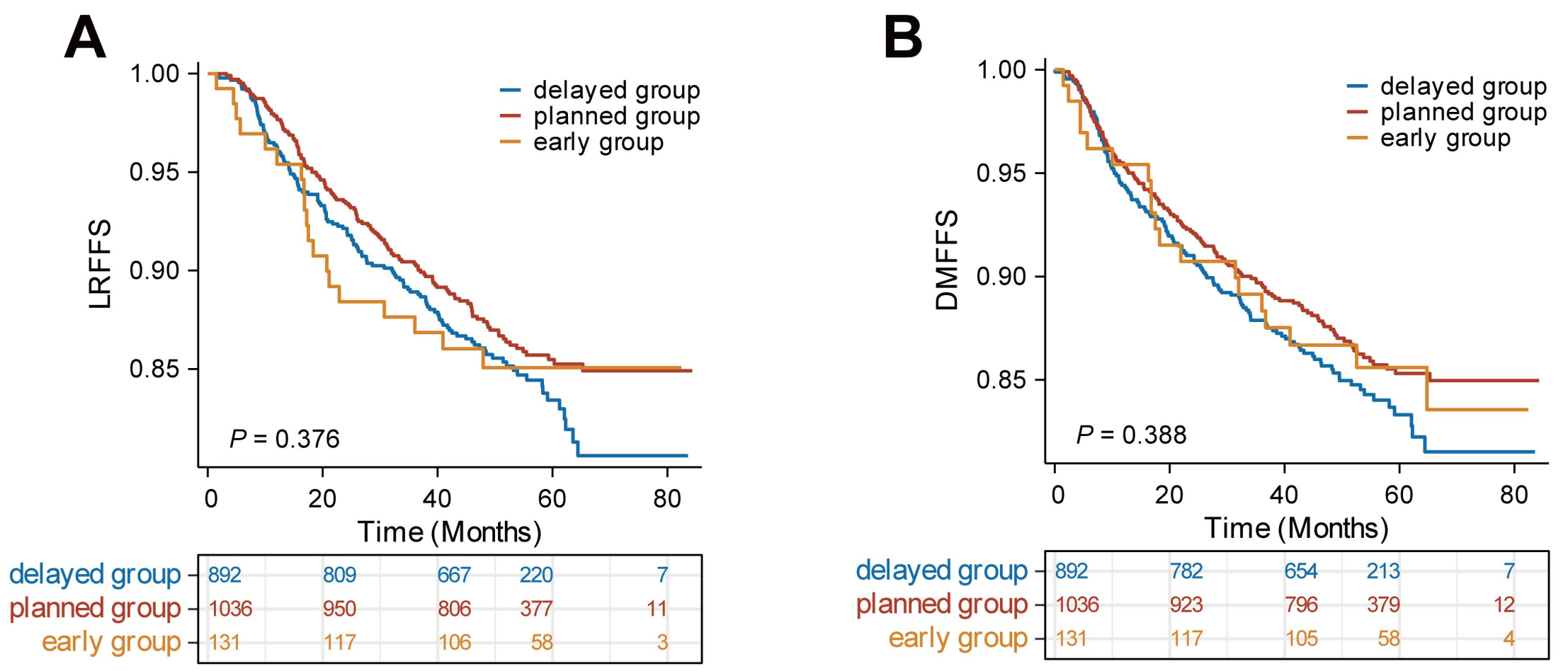

Groups	3-year survival (%)	5-year survival (%)
delayed group	88.79 (86.71–90.91)	83.42 (80.67-86.25)
planned group	90.33 (88.52-92.18)	85.25 (82.91-87.64)
early group	86.85 (81.21-92.88)	

Groups	3-year survival (%)	5-year survival (%)
delayed group	87.76 (85.59–89.99)	83.32 (80.60-86.13)
planned group	89.59 (87.71-91.50)	85.31 (83.00-87.69)
early group	88.34 (82.96-94.07)	83.56 (76.64-91.11)

Supplementary Fig. 3. Comparison of (A) LRFFS, (B) DMFFS probability between the planned completion group, the early completion group, and the delayed completion group. Abbreviations: Delayed group: the delayed completion group; Planned group: the planned completion group; Early group: the early completion group; LRFFS, locoregional failure-free survival; DMFFS, distant metastasis failure-free survival.(Relates to Fig. 3)

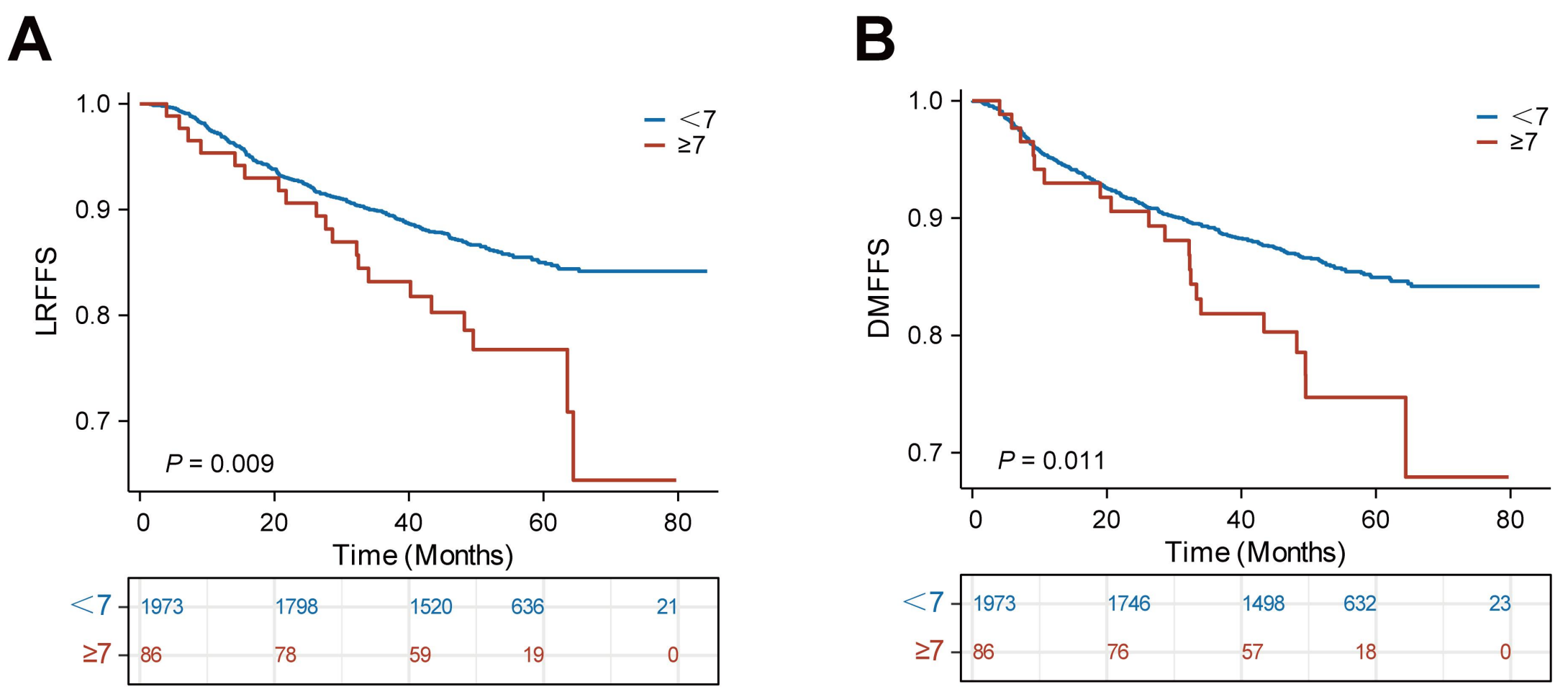

Groups	3-year survival (%)	5-year survival (%)
<7	89.77 (88.42-91.14)	84.86 (83.14-86.63)
≥7	83.19 (75.52-91.64)	76.77 (67.74-87.00)

Groups	3-year survival (%)	5-year survival (%)
<7	89.08 (87.68-90.49)	84.94 (83.23-86.69)
≥7	81.84 (73.92-90.61)	74.72 (65.24-85.58)

Supplementary Fig. 4. Kaplan-Meier survival curves for ΔRTT stratified by 7 days (A) LRFFS, (B) DMFFS. Abbreviations: RTT, radiotherapy treatment time; △RTT, the deviation in RTT; LRFFS, locoregional failure-free survival; DMFFS, distant

metastasis failure-free survival.(Relates to Fig. 4)

Variables	n (%)	△RTT< 7	△RTT≥7	OR (95%CI)	P	P for interaction
		No. of events/ No. of total				
All patients	2059 (100.00)	268/1973	20/86	1.93 (1.15 ~ 3.23)	**0.013**	
Gender						0.071
Male	1504 (73.05)	217/1437	14/67	1.49 (0.81 ~ 2.72)	0.201	
Female	555 (26.95)	51/536	6/19	4.39 (1.60 ~ 12.04)	**0.004**	
Age						0.995
≤45	767 (37.25)	73/738	5/29	1.90 (0.70 ~ 5.12)	0.206	
＞45	1292 (62.75)	195/1235	15/57	1.90 (1.04 ~ 3.50)	**0.038**	
Overall Stage						0.108
Stage I	51 (2.48)	1/50	1/1	NE	NE	
Stage II	222 (10.78)	11/213	1/9	2.30 (0.26 ~ 20.02)	0.452	
Stage III	938 (45.56)	81/900	5/38	1.53 (0.58 ~ 4.03)	0.388	
Stage IVa	848 (41.19)	175/810	13/38	1.89 (0.95 ~ 3.76)	0.072	
EBV DNA titer,copies						0.930
Negative	1473 (71.54)	150/1413	11/60	1.89 (0.96 ~ 3.71)	0.065	
Positive	586 (28.46)	118/560	9/26	1.98 (0.86 ~ 4.56)	0.107	
HGB, g/L						0.523
<113	528 (25.64)	74/504	7/24	2.39 (0.96 ~ 5.97)	0.061	
113-151	1441 (69.99)	189/1384	13/57	1.87 (0.99 ~ 3.53)	0.055	
>151	90 (4.37)	5/85	0/5	NE	NE	
Prescribed fractions						0.134
28-31	305 (14.81)	25/292	4/13	4.75 (1.36 ~ 16.52)	**0.014**	
32-36	1754 (85.19)	243/1681	16/73	1.66 (0.94 ~ 2.94)	0.081	
Residual or not						0.540
No	1673 (81.25)	218/1608	14/65	1.75 (0.95 ~ 3.22)	0.071	
Yes	386 (18.75)	50/365	6/21	2.52 (0.93 ~ 6.80)	0.068	
NLR						0.401
Normal	1053 (51.14)	122/1017	9/36	2.45 (1.12 ~ 5.32)	**0.024**	
Abnormal	1006 (48.86)	146/956	11/50	1.56 (0.78 ~ 3.13)	0.205	
Number of IC sessions						0.284
0	271 (13.16)	29/261	4/10	5.33 (1.42 ~ 20.02)	**0.013**	
≤3	1051 (51.04)	135/1005	10/46	1.79 (0.87 ~ 3.69)	0.115	
>3	737 (35.79)	104/707	6/30	1.45 (0.58 ~ 3.63)	0.428	
Interruption times						**0.033**
0	1018 (49.44)	146/1018	0/0	NA	NA	
1	645 (31.33)	72/620	9/25	4.28 (1.82 ~ 10.04)	**<0.001**	
＞1	396 (19.23)	50/335	11/61	1.25 (0.61 ~ 2.57)	0.537	
Treatment modality						0.079
RT alone	105 (5.10)	17/103	2/2	NE	NE	
IC + RT	493 (23.94)	69/471	5/22	1.71 (0.61 ~ 4.80)	0.305	
IC + RT＋AC	52 (2.53)	12/50	1/2	3.17 (0.18 ~ 54.57)	0.427	
CCRT	141 (6.85)	10/133	2/8	4.10 (0.73 ~ 23.02)	0.109	
CCRT＋AC	25 (1.21)	2/25	0/0	NA	NA	
IC + CCRT	1106 (53.72)	130/1059	10/47	1.93 (0.94 ~ 3.98)	0.074	
IC + CCRT＋AC	137 (6.65)	28/132	0/5	NE	NE	

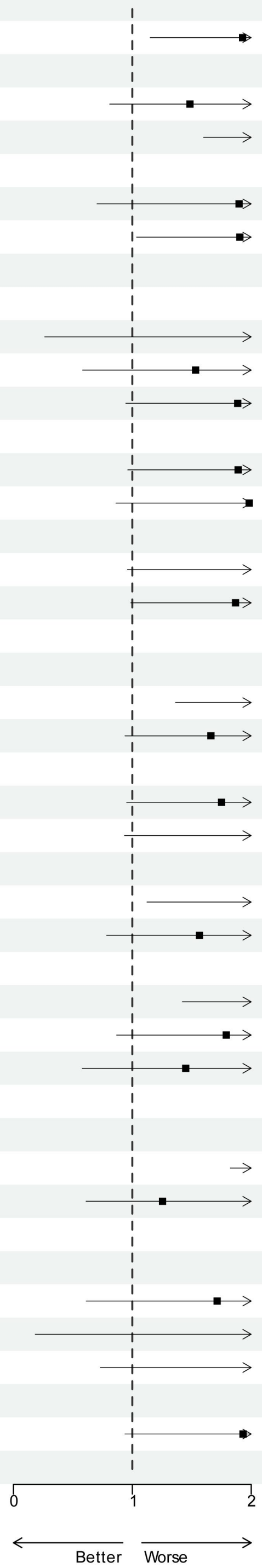

Supplementary Fig. 5. Subgroup analysis according to △RTT (<7 and ≥7)**.**(Relates to Table 3 and 4) NE, not estimable because of zero events or complete separation.

Supplementary Table S1. Univariate and multivariate analyses of overall survival.(Relates to Table 1)

Characteristic	Univariate analysis		Multivariate analysis	
	HR (95% CI)	P	HR (95% CI)	P
Gender				
Female	Reference		Reference	
Male	1.534 (1.148 - 2.050)	**0.004**	1.450 (1.077 - 1.952)	**0.014**
Age, year				
>45	Reference		Reference	
≤45	0.591 (0.456 - 0.767)	**< 0.001**	0.615 (0.471 - 0.803)	**< 0.001**
Overall stage (8th edition)				
I	Reference		Reference	
II	1.376 (0.308 - 6.146)	0.676	2.114 (0.427 - 10.455)	0.359
III	2.475 (0.609 - 10.056)	0.205	7.840 (1.745 - 35.216)	**0.007**
IV	6.530 (1.621 - 26.302)	**0.008**	19.305 (4.270 - 87.288)	**< 0.001**
EBV DNA titer, copies				
negative	Reference		Reference	
positive	2.129 (1.687 - 2.686)	**< 0.001**	1.956 (1.530 - 2.502)	**< 0.001**
HGB, g/L				
<113	Reference		Reference	
113-151	0.866 (0.669 - 1.121)	0.275	0.869 (0.661 - 1.141)	0.312
>151	0.318 (0.129 - 0.785)	**0.013**	0.599 (0.227 - 1.584)	0.302
Prescribed fractions				
28-31	Reference		Reference	
32-36	1.668 (1.136 - 2.448)	**0.009**	0.909 (0.592 - 1.396)	0.664
Treatment modality				

RT alone	Reference		Reference	
IC+RT	0.845 (0.511 - 1.399)	0.513	0.280 (0.157 - 0.501)	**< 0.001**
IC+RT+AC	1.506 (0.744 - 3.049)	0.255	0.397 (0.183 - 0.858)	**0.019**
CCRT	0.438 (0.213 - 0.903)	**0.025**	0.541 (0.245 - 1.196)	0.129
CCRT+AC	0.414 (0.097 - 1.779)	0.236	0.750 (0.157 - 3.574)	0.718
IC+CCRT	0.692 (0.429 - 1.118)	0.133	0.240 (0.139 - 0.414)	**< 0.001**
IC+CCRT+AC	1.078 (0.602 - 1.931)	0.800	0.331 (0.175 - 0.629)	**< 0.001**
Residual or not				
Yes	Reference			
No	0.977 (0.729 - 1.308)	0.874		
NLR				
Abnormal	Reference		Reference	
Normal	0.777 (0.616 - 0.979)	**0.033**	0.805 (0.637 - 1.016)	0.068
Number of IC sessions				
0	Reference			
≤3	1.192 (0.817 - 1.740)	0.363		
>3	1.394 (0.944 - 2.057)	0.095		
RTT, days				
>46	Reference		Reference	
≤46	0.707 (0.556 - 0.898)	**0.005**	0.795 (0.617 - 1.024)	0.076

Abbreviation: RTT, radiotherapy treatment time; △RTT: the deviation in RTT; EBV-DNA, plasma Epstein-Barr virus (EBV) DNA; HGB, hemoglobin; RT, radiotherapy; IC + RT, radiotherapy following induction chemotherapy; CCRT, concurrent chemoradiotherapy; IC + CCRT, induction chemotherapy plus concurrent chemoradiotherapy; NLR, neutrophil-to-lymphocyte ratio; AC, adjuvant chemotherapy; IC, induction chemotherapy.

Supplementary Table S2. Univariate and multivariate analyses of progression-free survival.(Relates to Table 1)

Characteristic	Univariate analysis		Multivariate analysis	
	HR (95% CI)	P	HR (95% CI)	P
Gender				
Female	Reference		Reference	
Male	1.554 (1.163 - 2.077)	**0.003**	1.446 (1.073 - 1.947)	**0.015**
Age, year				
>45	Reference		Reference	
≤45	0.621 (0.479 - 0.806)	**< 0.001**	0.661 (0.506 - 0.863)	**0.002**
Overall stage (8th edition)				
I	Reference		Reference	
II	1.385 (0.310 - 6.187)	0.670	2.013 (0.408 - 9.927)	0.390
III	2.526 (0.622 - 10.265)	0.195	6.982 (1.556 - 31.331)	**0.011**
IV	6.820 (1.693 - 27.471)	**0.007**	17.436 (3.862 - 78.726)	**< 0.001**
EBV DNA titer, copies				
negative	Reference		Reference	
positive	2.231 (1.768 - 2.815)	**< 0.001**	2.046 (1.599 - 2.617)	**< 0.001**
HGB, g/L				
<113	Reference		Reference	
113-151	0.899 (0.695 - 1.163)	0.417	0.893 (0.680 - 1.174)	0.418
>151	0.321 (0.130 - 0.792)	**0.014**	0.597 (0.226 - 1.576)	0.298
Prescribed fractions				
28-31	Reference		Reference	
32-36	1.688 (1.150 - 2.479)	**0.007**	0.892 (0.580 - 1.370)	0.601
Treatment modality				

RT alone	Reference		Reference	
IC+RT	0.877 (0.530 - 1.452)	0.610	0.316 (0.177 - 0.564)	**< 0.001**
IC+RT+AC	1.650 (0.815 - 3.341)	0.164	0.464 (0.215 - 1.003)	0.051
CCRT	0.452 (0.219 - 0.930)	**0.031**	0.565 (0.256 - 1.243)	0.156
CCRT+AC	0.421 (0.098 - 1.809)	0.245	0.766 (0.161 - 3.643)	0.737
IC+CCRT	0.732 (0.453 - 1.182)	0.202	0.280 (0.162 - 0.482)	**< 0.001**
IC+CCRT+AC	1.246 (0.696 - 2.232)	0.459	0.414 (0.219 - 0.784)	**0.007**
Residual or not				
Yes	Reference			
No	0.943 (0.705 - 1.263)	0.695		
NLR				
Abnormal	Reference		Reference	
Normal	0.778 (0.617 - 0.982)	**0.034**	0.804 (0.636 - 1.015)	0.066
Number of IC sessions				
0	Reference			
≤3	1.256 (0.861 - 1.833)	0.237		
>3	1.427 (0.967 - 2.106)	0.073		
RTT, days				
>46	Reference		Reference	
≤46	0.710 (0.559 - 0.902)	**0.005**	0.793 (0.616 - 1.022)	0.073

Abbreviation: RTT, radiotherapy treatment time; △RTT: the deviation in RTT; EBV-DNA, plasma Epstein-Barr virus (EBV) DNA; HGB, hemoglobin; RT, radiotherapy; IC + RT, radiotherapy following induction chemotherapy; CCRT, concurrent chemoradiotherapy; IC + CCRT, induction chemotherapy plus concurrent chemoradiotherapy; NLR, neutrophil-to-lymphocyte ratio; AC, adjuvant chemotherapy; IC, induction chemotherapy.

Supplementary Table S3. Univariate and multivariate analyses of locoregional failure-free survival.(Relates to Tables 3 and 4)

Characteristic	Univariate analysis		Multivariate analysis	
	HR (95% CI)	P	HR (95% CI)	P
Gender				
Female	Reference		Reference	
Male	1.553 (1.162 - 2.076)	**0.003**	1.471 (1.093 - 1.979)	**0.011**
Age, year				
>45	Reference		Reference	
≤45	0.596 (0.460 - 0.773)	**< 0.001**	0.624 (0.478 - 0.815)	**< 0.001**
Overall stage (8th edition)				
I	Reference		Reference	
II	1.369 (0.306 - 6.116)	0.681	2.036 (0.412 - 10.049)	0.383
III	2.481 (0.611 - 10.081)	0.204	7.681 (1.717 - 34.361)	**0.008**
IV	6.487 (1.610 - 26.131)	**0.009**	18.793 (4.175 - 84.592)	**< 0.001**
EBV DNA titer, copies				
negative	Reference		Reference	
positive	2.178 (1.726 - 2.749)	**< 0.001**	1.925 (1.507 - 2.460)	**< 0.001**
HGB, g/L				
<113	Reference		Reference	
113-151	0.881 (0.681 - 1.140)	0.336	0.876 (0.667 - 1.151)	0.342
>151	0.318 (0.129 - 0.784)	**0.013**	0.543 (0.206 - 1.426)	0.215
Prescribed fractions				
28-31	Reference		Reference	
32-36	1.666 (1.135 - 2.445)	**0.009**	1.013 (0.671 - 1.529)	0.951
Treatment modality				

RT alone	Reference		Reference	
IC+RT	0.843 (0.509 - 1.396)	0.508	0.273 (0.153 - 0.487)	**< 0.001**
IC+RT+AC	1.493 (0.738 - 3.024)	0.265	0.373 (0.173 - 0.803)	**0.012**
CCRT	0.440 (0.214 - 0.907)	**0.026**	0.558 (0.253 - 1.230)	0.148
CCRT+AC	0.409 (0.095 - 1.758)	0.230	0.790 (0.166 - 3.767)	0.767
IC+CCRT	0.702 (0.435 - 1.134)	0.148	0.236 (0.137 - 0.407)	**< 0.001**
IC+CCRT+AC	1.149 (0.641 - 2.057)	0.641	0.331 (0.175 - 0.627)	**< 0.001**
Residual or not				
Yes	Reference			
No	0.953 (0.712 - 1.276)	0.747		
NLR				
Abnormal	Reference		Reference	
Normal	0.776 (0.615 - 0.978)	**0.032**	0.816 (0.646 - 1.031)	0.088
Number of IC sessions				
0	Reference			
≤3	1.221 (0.837 - 1.782)	0.301		
>3	1.386 (0.939 - 2.045)	0.101		
△RTT, days				
<7	Reference		Reference	
≥7	1.810 (1.149 - 2.851)	**0.011**	1.874 (1.185 - 2.961)	**0.007**
Interruption times				
1	Reference			
0	1.135 (0.865 - 1.489)	0.362		
>1	1.286 (0.923 - 1.794)	0.138		

Abbreviation: RTT, radiotherapy treatment time; △RTT: the deviation in RTT;

EBV-DNA, plasma Epstein-Barr virus (EBV) DNA; HGB, hemoglobin; RT, radiotherapy; IC + RT, radiotherapy following induction chemotherapy; CCRT, concurrent chemoradiotherapy; IC + CCRT, induction chemotherapy plus concurrent chemoradiotherapy; NLR, neutrophil-to-lymphocyte ratio; AC, adjuvant chemotherapy; IC, induction chemotherapy.

Supplementary Table S4. Univariate and multivariate analyses of distant metastasis failure-free survival.(Relates to Tables 3 and 4)

Characteristic	Univariate analysis		Multivariate analysis	
	HR (95% CI)	P	HR (95% CI)	P
Gender				
Female	Reference		Reference	
Male	1.536 (1.149 - 2.052)	**0.004**	1.425 (1.058 - 1.919)	**0.020**
Age, year				
>45	Reference		Reference	
≤45	0.620 (0.478 - 0.804)	**< 0.001**	0.654 (0.501 - 0.854)	**0.002**
Overall stage (8th edition)				
I	Reference		Reference	
II	1.399 (0.313 - 6.249)	0.660	2.033 (0.411 - 10.053)	0.384
III	2.540 (0.625 - 10.322)	0.192	7.307 (1.633 - 32.700)	**0.009**
IV	6.885 (1.709 - 27.734)	**0.007**	18.691 (4.153 - 84.117)	**< 0.001**
EBV DNA titer, copies				
negative	Reference		Reference	
positive	2.210 (1.751 - 2.789)	**< 0.001**	1.979 (1.550 - 2.528)	**< 0.001**
HGB, g/L				
<113	Reference		Reference	
113-151	0.890 (0.688 - 1.152)	0.376	0.894 (0.680 - 1.174)	0.419
>151	0.321 (0.130 - 0.791)	**0.014**	0.568 (0.216 - 1.491)	0.250
Prescribed fractions				
28-31	Reference		Reference	
32-36	1.694 (1.154 - 2.486)	**0.007**	1.013 (0.671 - 1.530)	0.951
Treatment modality				

RT alone	Reference		Reference	
IC+RT	0.843 (0.509 - 1.396)	0.508	0.297 (0.166 - 0.529)	**< 0.001**
IC+RT+AC	1.493 (0.738 - 3.024)	0.265	0.430 (0.199 - 0.928)	**0.032**
CCRT	0.440 (0.214 - 0.907)	**0.026**	0.567 (0.257 - 1.252)	0.160
CCRT+AC	0.409 (0.095 - 1.758)	0.230	0.817 (0.171 - 3.910)	0.800
IC+CCRT	0.702 (0.435 - 1.134)	0.148	0.254 (0.148 - 0.438)	**< 0.001**
IC+CCRT+AC	1.149 (0.641 - 2.057)	0.641	0.373 (0.197 - 0.704)	**0.002**
Residual or not				
Yes	Reference			
No	0.962 (0.718 - 1.288)	0.793		
NLR				
Abnormal	Reference		Reference	
Normal	0.778 (0.617 - 0.981)	**0.034**	0.820 (0.649 - 1.036)	0.096
Number of IC sessions				
0	Reference			
≤3	1.243 (0.852 - 1.814)	0.260		
>3	1.434 (0.972 - 2.117)	0.069		
△RTT, days				
<7	Reference		Reference	
≥7	1.788 (1.135 - 2.817)	**0.012**	1.783 (1.128 - 2.818)	**0.013**
Interruption times				
1	Reference			
0	1.134 (0.864 - 1.487)	0.366		
>1	1.299 (0.932 - 1.812)	0.123		

Abbreviation: RTT, radiotherapy treatment time; △RTT: the deviation in RTT;

EBV-DNA, plasma Epstein-Barr virus (EBV) DNA; HGB, hemoglobin; RT, radiotherapy; IC + RT, radiotherapy following induction chemotherapy; CCRT, concurrent chemoradiotherapy; IC + CCRT, induction chemotherapy plus concurrent chemoradiotherapy; NLR, neutrophil-to-lymphocyte ratio; AC, adjuvant chemotherapy; IC, induction chemotherapy.
